# Understanding the Role of Extension Professionals in Public Health and One Health in Kansas

**DOI:** 10.3390/ijerph21060747

**Published:** 2024-06-07

**Authors:** Heather Poole, Antoinette Lona, Toni Rose M. Barroga, McKenzie Ghrist, Ellyn R. Mulcahy

**Affiliations:** 1 Master of Public Health Program, College of Veterinary Medicine, Kansas State University, Manhattan, KS 66502, USA; 2 Department of Diagnostic Medicine and Pathobiology, College of Veterinary Medicine, Kansas State University, Manhattan, KS 66502, USA; 3 Department of Agriculture, Bureau of Animal Industry, Quezon City 1100, Philippines

**Keywords:** community education, community engagement, collaboration, cultural awareness

## Abstract

This study aims to understand the roles of selected extension professionals (EPs) in the field of public health and One Health and the challenges involved in performing these duties to their communities in the state of Kansas. To evaluate the role of EPs in public health and One Health, researchers interviewed nine (9) EPs following a set of structured questions. Emerging themes were extrapolated from the responses of the EPs. Researchers assigned codes for qualitative analysis and assigned themes related to public health, One Health, and effective delivery of services. Researchers identified the following themes related to the role of EPs in public health (youth development, physical activity, personal health care, proper nutrition, access to transportation), One Health (food safety and food security, environmental health, disease control and prevention) and effective delivery of services (community engagement, collaboration, challenges in implementation). The study provided an overview of the diverse roles that EPs play in public health and One Health, keys on how to engage the community effectively, and challenges in extending services to the community.

## 1. Introduction

Extension professionals (EPs), also referred as extension agents, are community-based educators who serve farmers and other residents of rural communities, including other people living in urban areas [1]. EPs are typically assigned to serve one county or geographic area of counties and provide non-formal education and learning activities related to agriculture, health and well-being, and youth development, among others [2,3]. In the agriculture field sector, EPs are well recognized to be a vehicle of information to ensure that the findings of research initiatives are used to improve farmers’ productivity and meet the needs and demands. Globally, it is estimated that there are at least 800,000 EPs, with more than 90% located in the developing world [4,5,6].

In the United States (US), the Cooperative Extension System (CES) is operated through the land grant university (LGU) system, in collaboration with federal, state, and local governments. It aims to improve the health outcomes and livelihoods of the communities [7]. The CES houses a team of campus-based subject matter specialists and support county-level EPs whose activities are driven by the needs and priorities of the local communities, including the available resources from the sponsoring LGUs [8,9].

LGUs are tasked not only with performing research and teaching, but also with conducting extension. The mission of extension is “Taking the University to the People”; thus, EPs apply a grassroots approach through partnership with different community stakeholders [10]. As the research findings of LGUs are extended to the communities, EPs are recognized to be a credible source of science-based information to the public [11]. EPs have a strong and long history in serving the community over the past 100 years as supported by three federal laws—(1) the Morrill Act of 1862, which provided for the establishment of agriculture colleges and ensured funding and continuous promotion for agricultural education, production, and productivity [12]; (2) the Hatch Act of 1887, which provided funds for LGUs to establish agricultural stations, thereby allowing agriculture faculty to conduct field demonstrations and offer short courses for farmers; and (3) the Smith–Lever Act of 1914, which created the CES, which was envisioned to share practical applications for farmers and homemakers from the research activities conducted in LGUs [13]. During the 1930s Great Depression in the US, EPs assisted farmers in organizing themselves into cooperatives so they could easily market their agricultural produce [14].

The education efforts of EPs are primarily focused in four areas: (1) agriculture and natural resources, (2) youth development, (3) family and consumer sciences, and (4) community development [9]. Over the years, the role of EPs in the community has evolved to disaster management and response, hurricanes, oil spills, wildfires, and zoonotic diseases. Recognizing that humans, animals, and the environment are inter-dependent in addressing disease concerns, applying a One Health approach fits in the role of EPs as they serve as a bridge to human healthcare, animal husbandry, and environmental concerns [15]. EPs also work along with frontline responders to assist in several capacities in emergency management services and public health departments [16,17]. Further, EPs were documented to be the frontline responders during the 2004 Florida hurricane season, when they assisted in providing food and water, organizing chainsaw crews, and helping secure electrical generators to the public [18]. Recently, with the emergence of the COVID-19 pandemic and limited interactions among people, EPs adopted the “new normal” and developed alternative methods to serve the changing needs of the community. In China, the agriculture department created a platform linked to the National Cloud Platform for Grassroot Agricultural Technology Extension to mitigate the effects of the pandemic on farmers by offering online expert consultation during spring plowing, promoting online market information in selecting crops to maximize cost, and disseminating prevention and control measures regarding fall armyworm (a highly infectious pest devastating crop fields) [19]. A study in Utah showed that EPs provided online learning activities for youth, communicating with local learners on how EPs can support the needs of the residents, and forging partnerships with other governmental organizations for a more coordinated effort during the height of the COVID-19 pandemic [20].

In the state of Kansas, the Kansas State University Research and Extension is present in all its 105 counties. This program has county extension offices strategically located in different areas of Kansas, experiment fields, area extension offices, and regional research centers across the state [21].

The study aimed to explore the role of EPs in the state of Kansas. In particular, the researchers investigated how EPs perform their duties as they relate to public health and One Health and sought to understand the challenges in implementing extension activities.

## 2. Materials and Methods

The study was divided into three (3) phases of implementation: (1) selection of participants; (2) data collection through an online survey questionnaire and face-to-face/online interview approach; and (3) data analysis.

### 2.1. Phase 1: Selection of Participants

Using the database of the Kansas State University–Master of Public Health Program of students’ previous field experience preceptors or locations for the past five years, potential participants were contacted through electronic mail to explain the purpose of the study and were invited to participate by completing the questionnaire enclosed in the electronic mail. A structured online questionnaire link created through Qualtrics^XM^ was sent to 22 potential participants of this study. A follow-up was sent to potential participants after two weeks to complete the questionnaire. The online questionnaire link was closed two weeks after the follow-up email was sent. The institutional review board of Kansas State University (IRB#9802) approved the study protocol, and researchers obtained informed consent from all participants.

### 2.2. Phase 2: Data Collection: Online Survey Questionnaire and Face-to-Face/Online Interview Approach

The structured questionnaire was designed to gather information from the respondents regarding the following sections—(1) participant’s demographic information and (2) roles in the public health workforce. A separate face-to-face interview was also organized for all participants who completed the questionnaires. Questions were focused on further understanding about their community involvement as EPs and how public health is integrated into their daily work activities. The first author conducted all in-person interviews (approximately 30 min each) to each EPs, and researchers transcribed the answers to each question.

### 2.3. Phase 3: Data Analysis

**Figure 1:**
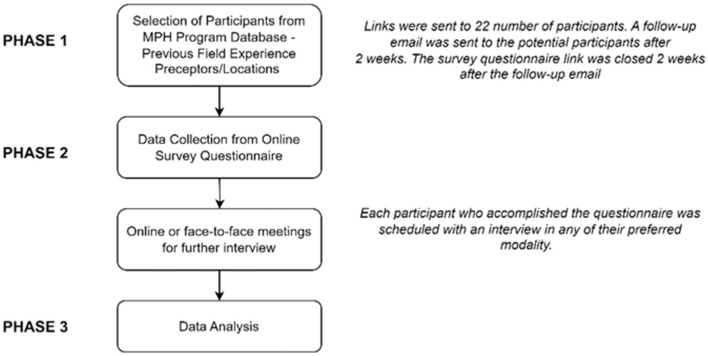
Schematic diagram of the study steps.

Quantitative and qualitative data were collected from the participants through the questionnaire and interview. The participants’ responses in the online questionnaire were extracted and summarized from Qualtrics^XM^. (Qualtrics, Seattle, WA, USA, 2022) Likewise, researchers transcribed answers from the interviews and written interview transcripts were reviewed by the first and corresponding author immediately after each interview to ensure the content’s accuracy. The transcripts and notes were reread independently by all authors. For accurate coding of the data, the authors discussed and confirmed the identified recurring patterns and emerging themes. The corrected, typed transcripts and notes were used for analysis. NVivoR1 software (QRS International Ltd., Burlington, MA, USA, 2020) was utilized to classify, sort, and analyze the data. Thematic analysis was used to assess the responses. Figure 1 shows the schematic diagram of the processes undertaken in this study.

## 3. Results

### 3.1. Participants’ Profile

The study gathered responses from nine (9) participants working in different counties in Kansas. Participants were mostly female (88.9%) and Caucasian (66.7%). All participants were working in county extension offices, particularly in urban (66.7%) counties. A majority of the EPs were working in nutrition (55.6%). Table 1 shows the demographics of the participants in this study.

### 3.2. Role of EP in Public Health

There were five (5) emerging themes identified related to EPs’ role in public health—proper nutrition, personal healthcare, youth development, physical activity, and transportation. Most of the EPs (89%) explained their role in providing guidance related to eating and cooking healthy foods and access to healthy food resources. One EP explained, “*For us, we wanted to teach people cooking skills. We believe health starts individually*”. One of the respondents shared that promoting the practice of healthy eating habits in schools is critical so that children will grow up with better eating habits. The EP mentioned in the interview that “*To be healthy, you must eat right. It starts with school. If children learn to eat right when they are younger, they will likely grow up being healthier*”. EPs also highlighted the relevance of their Supplemental Nutrition Assistance Program (SNAP) and Supplemental Nutrition Assistance Program Education Program (SNAP-Ed) grants as they can give nutritious foods to low-income individuals with limited resources as well as educate them on how to choose and prepare healthy foods. EPs discussed the necessity of increasing nutrition-based education in their communities and its role in promoting food security.

EPs also impart their knowledge on how to practice proper personal healthcare. Three respondents (33%) discussed various roles in how they help their community on how to properly take care of themselves. One respondent cited that “*Most farmers are men, and they are poor at taking care of their health. I use myself as an example to try to get men to see doctors*”. The respondent also added, “*Farmers think that they are tough, so I remind them to drink water in the summer. I also teach them how to have hearing protection and how to be safe in using power tools*”. Another respondent also discussed the approach how to make public health very personal—“*I teach about handwashing. My focus is the consumers and grab their understanding of public health by making it personal and keeping yourself healthy*”.

Thirty-three percent (33%) of the respondents mentioned their assistance in youth development activities, particularly 4-H activities. The 4-H (Head, Heart, Hands, and Health) program is the premier youth development program of the US Department of Agriculture (USDA), which aims to promote positive development and facilitate learning among youth in the fields of science, engineering, citizenship, and healthy living [22]. Respondents discussed how EPs are busy during the summer months preparing for 4-H activities such as camps and county fairs. During 4-H activities, they also educate youth about nutrition, so that the community can produce healthy individuals. Meanwhile, two respondents (22%) explained that along with proper nutrition, they also promote physical activities. One respondent said, “*We need to eat healthy and move more*”. While only one respondent (11%) pointed out their assistance with transportation, it is noteworthy to discuss how EP is bringing this concern—“*We help people find transportation to meet their working needs*”. Table 2 shows the summary of findings of the role of EPs in public health.

### 3.3. Emerging Role of EP in One Health

The role of EP in One Health emerged as a theme during interviews. Three respondents (33%) disclosed their experiences on how One Health applies to their role as EPs. Three themes emerged from the interviews with the EPs—food safety, environmental health, and disease control and prevention.

Respondents defined their role in food safety and food security by training their community to produce healthy foods through proper home horticulture, pesticide safety, and good animal husbandry practices—“*Most of my time is spent in having one-on-one education in helping homeowners about home horticulture and farmers about agriculture and how to have a productive farm*”. Meanwhile, two respondents (22%) talked about how EPs contribute to responding to some environmental issues to the community—“*I responded to a poison ivy situation when a daycare called me about this concern. I was also involved in snake identification and bee stings*”. Two EPs (22%) tackled how they carry out activities to prevent and control diseases in both humans and animals. One respondent noted that writing articles in newspapers for insect bite prevention is one of the regular activities contributed to One Health. Respondents conveyed that they teach about vector-borne diseases and bed bugs, including appropriate medications for livestock. To inform their constituents about the health department’s activities, EPs also collaborate with health departments to be updated about vaccine schedules. Table 3 shows the summary of findings on the role of EPs in One Health.

### 3.4. Effective Delivery of Services for EPs

During interviews, researchers found recurring themes in how EPs deliver extension services to the community. Three emerging themes were identified—community engagement, collaboration, and challenges in implementation.

Community engagement is central to the role of EPs. Most of the respondents (89%) stressed how reaching out and creating conversations with the members of the community is critical to understanding their concerns. One respondent cited “*We need to be more aware of the things going on with the community. Networking helps with the job since you want to know people and people need to know you as well*”. Another respondent commented on how they promote their activities in church, clinics, and pantries so they can reach out to more people. One EP clarified that “*Extension is meeting the community needs. We help people in the community have the access to resources they need*”. A respondent also added, “*It is important to know your subject by knowing how to research the current knowledge*”.

One EP cited how reading local news columns and weekly news reports can help them be informed on the issues and concerns around their community and actively assess the resources that will be beneficial to the people. An EP emphasized the importance of EP as it relates to public health—“*I am a community educator, and my hours are for the community. It is not a 9–5 Monday to Friday job because public health is 24/7*”.

Meanwhile, collaboration is also an emerging theme among all EPs as they expound on how they partner with organizations, and health departments to help in activities such as providing healthcare services and materials for schools and creating policies for their community. One EP also mentioned how they work along with the Women Infant Children (WIC) program and senior programs and participate in health fairs and food pantries, among others, so they can have a more strategic approach in addressing community needs—“*We do a lot of strategic efforts in extension. We must understand each other’s department*”.

In helping the community, the interviews among EPs also revealed some challenges in implementing their line of work. Four (4) sub-themes emerged from this major theme—culture awareness, academic-extension gap, misinformation/lack of information, and adaptation to change. It is interesting to note how one EP stressed that language has been a barrier to extending help to the community. “*We need to be bilingual. We have no personnel who can understand the Latino language. We need cultural awareness because I accidentally offended someone because I did not know about his culture*”. Another EP also disclosed how communication is important in their role due to the vast roles they play in their community—“*We deal with a variety of people—community, producers, and different ethnic backgrounds*”. Two EPs also observed the importance of universities/campus specialists; however, one EP explained believes some researchers apply for some grants, but this is not what the community needs. Another EP shared an observation on how misinformation and lack of basic science knowledge cause a challenge in helping people improve their quality of life. Almost half of the EP (44%) identified how “generational gaps” between new graduates and experienced EPs are becoming a challenge in delivering their services—“*There is a huge gap between those coming out of school and those being in the [extension] for 30 years. Being able to adapt to changes and make ourselves relevant is important in extension*”. Challenges in adapting to change were also supported by two EPs in their interviews—“*There is a common thread in extension that this is the way [we did it] and people are not willing to change*” and “*With baby boomers, change is okay. But this is the way we did it so there is need to change it*”. Table 4 summarizes the findings of the role of EPs in effective delivery of services.

## 4. Discussion

The extension work model was envisioned to connect subject matter experts in LGUs, so they can train extension agents or EPs, who will thereby share this knowledge or skill with their community members. Since EPs live within the same community, they fully understand and experience the current social, economic, and environmental challenges [11].

In this study, the findings described how EPs perform a wide array of activities. While extension work is mostly focused on nutrition, agriculture, and youth development, the interviews revealed how EPs also act as agents to promote public health and One Health by responding to the needs of the community in animal disease control and prevention, environmental health concerns, food safety, and personal healthcare. Moreover, EPs also identified the relevance of understanding community needs through promoting programs and creating networks among community members. Through conversations, EPs can deeply evaluate pressing issues in their community and address their concerns strategically. Various methods of solving community problems such as teaching technical skills and knowledge, collaborating with different departments/organizations, and finding ways for some day-to-day concerns such as access to nutritious foods and even transportation are evident among all the EPs’ responses. Recent research in Kansas has shown how public health professionals are concerned with different pressing issues in their community such as funding, equitable access to transportation, affordable healthcare, political interference in public health concerns, and the need for cultural competence to ensure equitable care [23]. This reinforces the similarities experienced by EP in the current study.

In the interviews, EPs emphasized how they educate about proper diet and nutrition, home gardening, farm productivity techniques, livestock breeding and medications, and cooking, among others. EPs are exploring options on how the community can access nutritious foods and forge partnerships with schools and other government programs to ensure wider promotion of their programs. Several respondents also explained the funding they receive from federal grants (e.g., SNAP, SNAP-Ed), which shows how EPs seek several ways to generate more funding to operationalize their programs. US federal programs such as SNAP, according to studies, show considerable success in reducing poverty and food insecurity and are linked to improved health [24]. As communities devise alternative and creative methods for delivering accurate information to the public, the expertise of EPs can be utilized, as they are a trusted source of reliable information that can illustrate appropriate practices with consistent guidance [4]. EPs serve as an important conduit for reliable agricultural information and technology which will be beneficial to farmers [6].

While all the abovementioned practices fall under the portfolio of nutrition and food safety, these case examples also demonstrate how EPs integrate the major pillars of food security into their roles. Food security is a multi-dimensional concept characterized by the four pillars of availability, access, utilization, and stability [25]. A qualitative study was conducted among EPs globally to analyze how nutrition is integrated into extension services. Results showed that EPs’ practices can be further disaggregated into these three pillars—food availability (home gardening, effective farming techniques, reduction of post-harvesting techniques, breeding animals for protein sources); food access (enhanced marketing strategies for nutrient-rich vegetables, increased availability of missing sources of nutrition); food utilization (recipes and food preparation techniques that maximize nutritional benefits) [4]. EPs go beyond transferring knowledge and skills to ensuring that nutritious foods are also available, accessible, and utilized.

Through the lens of the One Health approach, which brings together animal, human, and environmental health concerns, the most effective way to address zoonotic diseases is through communication and education [26,27]. EPs can play a role in addressing disease prevention and control because of their credibility and local cultural knowledge in their respective communities [28,29,30].

Respondents highlighted how community engagement through joining different local activities can allow EPs to interact with people and understand the needs of the local community. The goal of extension education programming is to create impact through individual behavioral change and adoption of best practices [31,32]. A recent study reported that the transtheoretical model of change can be useful for EPs to formulate appropriate messaging and supporting activities based on the current stage of an individual or local audience. Therefore, reaching out to community members will be helpful for EPs, as this will provide them opportunities to know their audience’s perceived barriers and suggest specific changes during the process of behavior change [33].

However, to be able to deliver all these services effectively, EPs are also faced with numerous challenges in their field. LGUs and CES receive inadequate funding for their programs to accomplish their purpose of serving the people. Due to federal and state funding reductions, LGUs have more competitors with other public and private institutions. This creates a shift from the earlier mission of LGUs as a “people’s university” towards a focus on costly research and graduate and undergraduate training [13,34]. This supports the observation of a respondent regarding the misalignment between the funding grant of the LGU and what the community needs.

Meanwhile, some studies also identified that responding to a rapidly diversified stakeholder population, historically underserved and marginalized communities of color is becoming a challenge to the role of EPs [35,36,37,38]. With the growing number of immigrants in the US population (approximately 13.7% of the nation’s population in 2018) [39], studies have shown that there is an increasing awareness to adopt an intercultural competence framework in the professional curriculum for EPs to equip them with competencies that will better serve the increasingly diverse clientele [40,41]. This was demonstrated in this study, in which one EP explained that they lost some clients since they were not knowledgeable in speaking Spanish. Different organizations offer training in cultural competence using various modalities (video/discussion series, state tour programs, and cross-cultural immersion. Participants who watched the training video disclosed that the material helped increase their awareness about diversity. Meanwhile, another study reported that EPs who joined the immersion program in a Latin American country for two weeks mentioned that their cultural appreciation and empathy increased based on their first-hand experience [42,43,44]. However, they also cited that this can be applied to their current line of work, as they experience difficulties in the recruitment of diverse participants. The additional goals, increasing responsibilities, and changing trends warrants the need for EPs to be equipped with a diverse set of capacities to respond effectively [45].

Limitations to our study include the small sample size and low response rate (41%) of EPs and the boundary of the geographical area to Kansas. This figure is most similar to the results of a meta-analysis study which showed a 44.1% response rate to online surveys conducted in education-related research [46]. Future studies should include more EPs in more counties in Kansas and other states. Findings from our study indicate that EPs in Kansas are an essential component of the public health network in the state, especially when it comes to community education and outreach.

## 5. Conclusions

This pilot study, albeit with limited number of participating EPs, underscored significant insights to understand the diverse role of EPs in extending service to the community. Central to the duties of EPs is to protect and promote public health and apply One Health where it may be deemed relevant.

## Figures and Tables

**Table 1:** Demographics of participants.

	Responses (%) *N* = 9
Race	
Caucasian	6 (66.7)
African American	1 (11.1)
Not reported	2 (22.2)
Type of County *	
Frontier	1 (11.1)
Semi-urban	2 (22.2)
Urban	6 (66.7)
Nature of Work	
Agriculture and Natural Resources	2 (22.2)
Nutrition	5 (55.6)
Family and Consumer Sciences	3 (33.3)

* Kansas County peer designations.

**Table 2:** Summary of findings regarding the role of EPs in public health.

Theme	Summary of Findings	Frequency ^1^ (# ^2^, % ^3^)
Youth Development	4-H-related activities are facilitated to encourage more youth in activities such as livestock raising in preparation for summer county fairs.	3 (3, 33%)
Physical Activity	EPs educate the community to engage in more physical activities, along with eating a proper diet.	2 (2, 22%)
Personal Healthcare	Areas of emphasis including drinking water in the summer, hearing protection and safety around power tools, handwashing, and dining with diabetes are imparted by EPs to the community.	5 (3, 33%)
Promotion of Proper Nutrition	Several counties have grants for SNAP and SNAP ED which allow EPs to deliver services related to proper nutrition, food assistance programs, and access to healthy foods. Eating nutritious foods is one of the keys to having a healthy body. This must be learned in schools so that children will likely grow up having good health habits.	10 (8, 89%)
Access to Transportation	EP is helping to facilitate the needs of people to find transportation to meet working needs	1 (1, 11%)

^1^ Number of times the theme was mentioned by the respondent; ^2^ Number of respondents who mentioned the theme; ^3^ Percentage of respondents who mentioned the theme.

**Table 3:** Summary of findings of the role of EPs in One Health.

Theme	Summary of Findings	Frequency ^1^ (# ^2^, % ^3^)
Food Safety and Food Security	EPs assist farmers and homeowners on how to have productive farming, including sharing proper animal husbandry practices and an overview of the food industry.	5 (2, 22%)
Environmental Health	EP has experiences related to the needs of the community on bee stings, snake identification, and poison ivy prevention. EPs allow the community to understand the entire food supply chain and its effect on the environment.	4 (2, 22%)
Disease Control and Prevention	EP writes articles related to insect bite prevention, vector-borne diseases, and bed bugs. Agriculture services related to medications in livestock are also performed. Collaboration with health services is conducted to update the community about the vaccine dates.	5 (2, 22%)

^1^ Number of times the theme was mentioned by the respondent; ^2^ Number of respondents who mentioned the theme; ^3^ Percentage of respondents who mentioned the theme.

**Table 4:** Summary of findings on understanding the role of EPs in effective delivery of services.

Theme	Summary of Findings	Frequency ^1^ (# ^2^/% ^3^)
Community Engagement	Understanding the needs at the grassroots level is important. EPs help the community by helping to bridge the gap between their needs and finding solutions to have access to these resources Active promotion of activities in health clinics, churches, and common food pantries and creating conversation among community members can also help in finding out the needs of the community. The job does not have a 9–5 Monday to Friday schedule because public health is 24/7.	18 (8, 89%)
Collaboration	Collaboration with health departments, and organizations in creating policies and programs relevant to women, infants, children, and seniors. Participation in food fairs, health fairs, food pantries, and youth programs to promote activities in public health.	4 (3, 33%)
Challenges in Implementation		
Academe-Extension Gap	Universities play a vital role in bridging the gap between academics’ research activities and community needs. Sometimes, the funding of the researcher is not what the community needs.	3 (2, 22%)
Culture Awareness	EPs must be “culturally aware” to better assist the community. Learning about different ethnic backgrounds and their language has been a challenge for some EPs.	4 (1, 11%)
Misinformation/Lack of Information	Misinformation and lack of knowledge about general science also hampers the delivery of services by EPs	3 (1, 11%)
Adaptation to Change	Generation gaps and adapting to change are posing a challenge in implementing their work	7 (4, 44%)

^1^ Number of times the theme was mentioned by the respondent; ^2^ Number of respondents who mentioned the theme; ^3^ Percentage of respondents who mentioned the theme.

## Data Availability

Data is available from E.R.M. upon request.
